# [Corrigendum] Human cytomegalovirus RL13 protein interacts with host NUDT14 protein affecting viral DNA replication

**DOI:** 10.3892/mmr.2026.14000

**Published:** 2026-08-24

**Authors:** Guili Wang, Gaowei Ren, Xin Cui, Zhitao Lu, Yanping Ma, Ying Qi, Yujing Huang, Zhongyang Liu, Zhengrong Sun, Qiang Ruan

Mol Med Rep 13: 2167–2174, 2016; DOI: 10.3892/mmr.2016.4778

Following the publication of the above article, a concerned author drew to the Editor's attention that there appeared to be splicing events (and breaks in the continuity) of certain of the gels shown for the GST-pulldown experiments in Fig. 1 on p. 2170, and the co-immunoprecipitation experiments shown in Fig. 2 on p. 2171. After assessing their data, the authors have responded to the office to explain that they integrated data from repeated analyses into these figures, rather than the data all being generated from the same experiments. The authors also presented all their original data to the Editorial Office for our perusal, and wished to point out that the data in question were only intended to show qualitative results, and did not form part of the main analysis of the study; nor did the results from these experiments have a major impact on the reported conclusions.

After volunteering to repeat these experiments, the authors were given authorization to do so by the Editor, and the revised versions of Figs. 1 and 2 (with revised figure legends) are shown opposite. The revised figures have both resolved the presentational difficulties associated with the original figures, and confirmed the original findings as presented in the published paper. All the authors approve of the publication of this corrigendum, and are grateful to the Editor of *Molecular Medicine Reports* for allowing them the opportunity to publish this; furthermore, they apologize to the readership for any inconvenience caused.

## Figures and Tables

**Figure 1.**
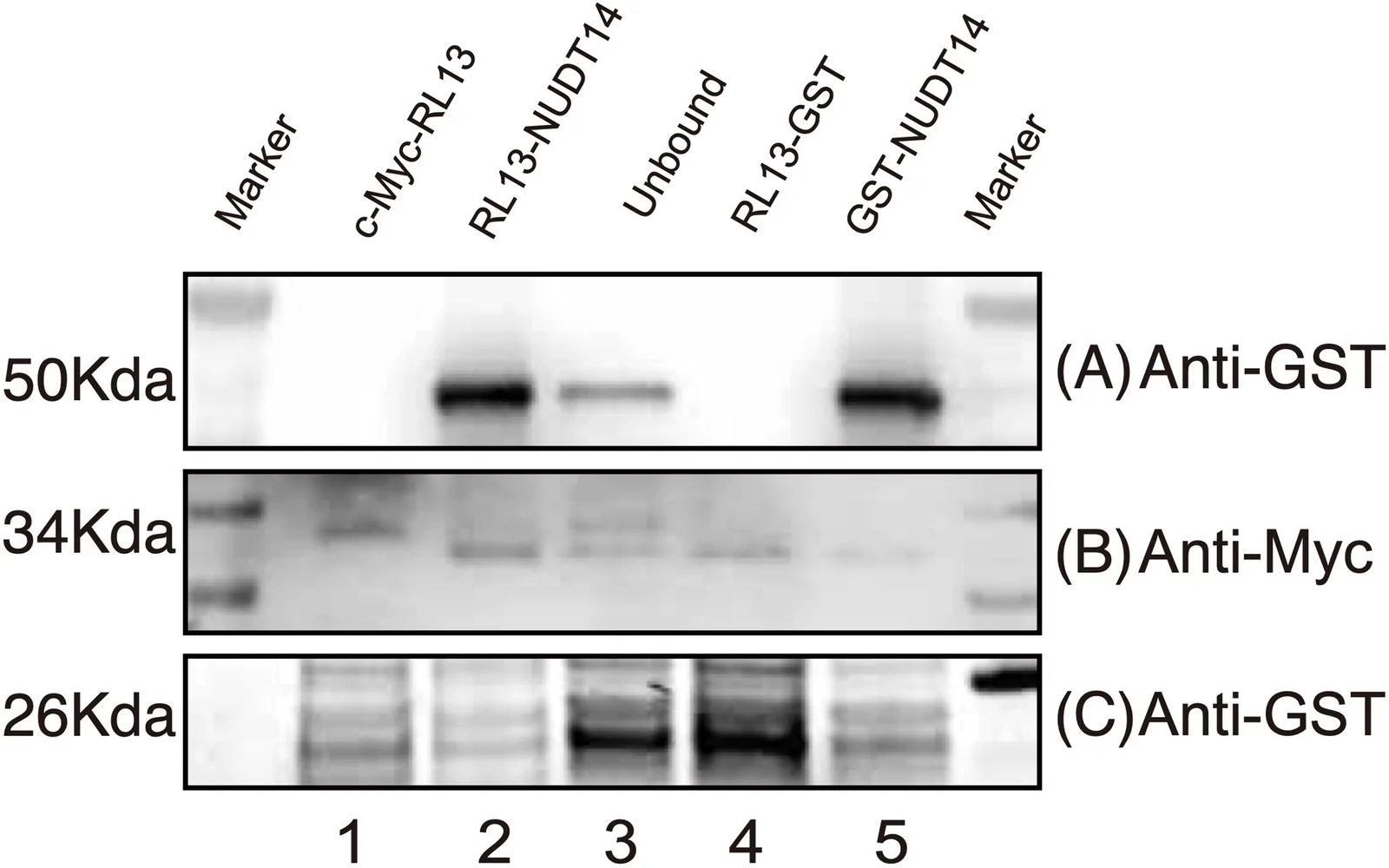
Direct interaction between NUDT14 and RL13 was analyzed by GST pull-down assay. GST-tagged NUDT14 served as bait, c-Myc-tagged RL13 (an HCMV glycoprotein) as prey, and free GST as negative control. Products were resolved on three membrane strips and detected with (A) anti-GST (~50 kDa of GST-tagged NUDT14), (B) anti-c-Myc (~34 kDa of c-Myc-tagged RL13), or (C) anti-GST (~26 kDa of free GST). Lanes, left to right: Marker; lane 1, c-Myc-RL13; lane 2, GST-NUDT14 pull-down; lane 3, unbound flow-through; lane 4, free-GST control pull-down; lane 5, GST-NUDT14; Marker. (A) GST-NUDT14 fusion. Strong bands in lanes 2 and 5 confirm bait enrichment and expression; a weak band in lane 3 is residual uncaptured protein; lanes 1 and 4 are negative. (B) c-Myc-RL13 (prey). Lane 1, input control. Lane 2, c-Myc-tagged RL13 pulled-down by GST-NUDT14, indicating a physical interaction; lane 3, unbound c-Myc-taggedRL13; lane 4 is free-GST control, showing a faint signal. (C) Free GST confirms bait in the control system. Band in lane 4 is the strongest one; faint bands elsewhere are GST degradation or background. GST, glutathione S-transferase; NUDT14, nucleo-side diphosphate-linked moiety X (nudix)-type motif 14.

**Figure 2.**
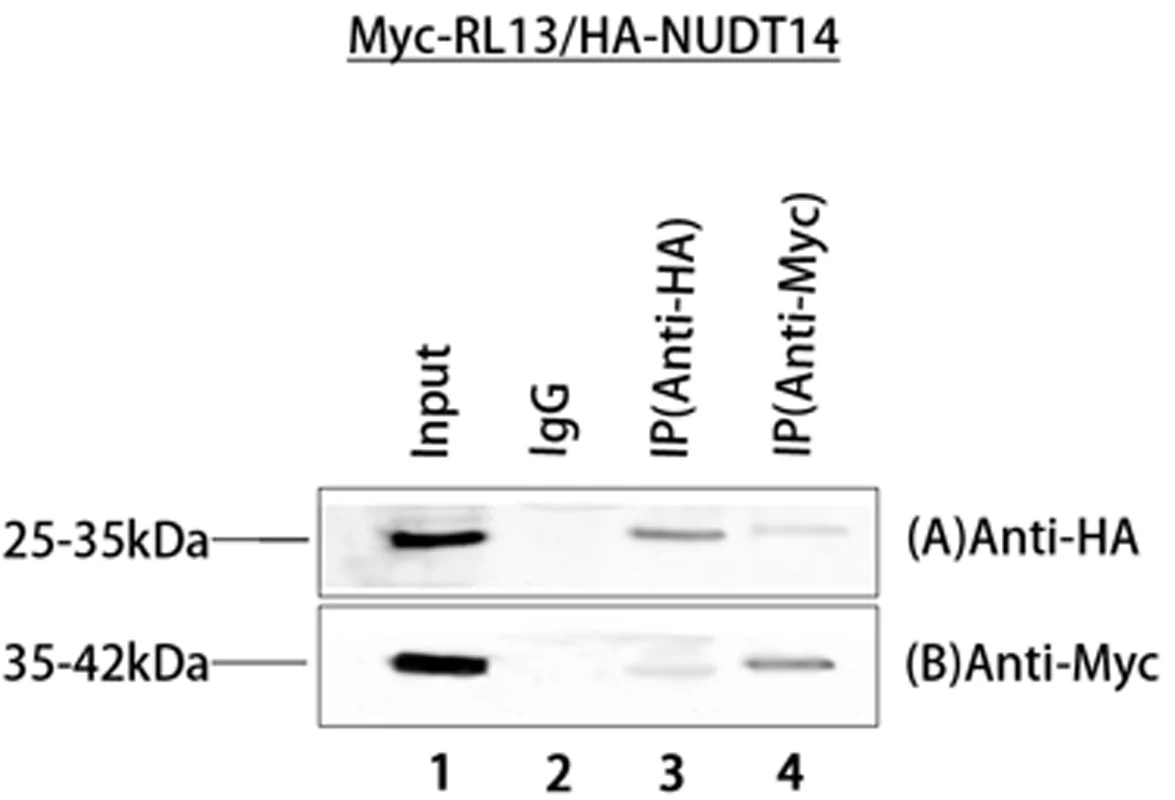
The interaction between c-Myc-labeled RL13 and HA-labeled NUDT14 was examined by co-immunoprecipitation (Co-IP). 293T cells were transiently co-transfected with pCMV-Myc-RL13 and pCMV-HA-NUDT14 plasmids. At 48 h post-transfection, whole-cell lysates were prepared and immunoprecipitated (IP) with normal rabbit IgG (negative control, lane 2), anti-HA antibody (lane 3), or anti-Myc antibody (lane 4). Whole-cell lysates were used as the input control (lane 1). The precipitates and inputs were resolved by SDS-PAGE and immunoblotted (IB) with anti-HA (panel A) or anti-Myc (panel B) antibodies.

